# Supplementary material for: Ligand-tethered lipid nanoparticles for targeted RNA delivery to treat liver fibrosis
Source: Nat Commun. 2023 Jan 17;14:75. doi: 10.1038/s41467-022-35637-z (PMC9845313; doi:10.1038/s41467-022-35637-z)
Supplement: Supplementary file 1 — Supplementary Information [file 41467_2022_35637_MOESM1_ESM.pdf]

Supplementary Materials for

Ligand-Tethered Lipid Nanoparticles for Targeted RNA Delivery to Treat Liver Fibrosis

Xuexiang Han, Ningqiang Gong, Lulu Xue, Margaret M. Billingsley, Rakan El-Mayta, Sarah J. Shepherd, Mohamad-Gabriel Alameh, Drew Weissman, Michael J. Mitchell*

*Corresponding author. Email: mjmitch@seas.upenn.edu (MJM)

This PDF file includes:

Figs. S1 to S24
Table S1

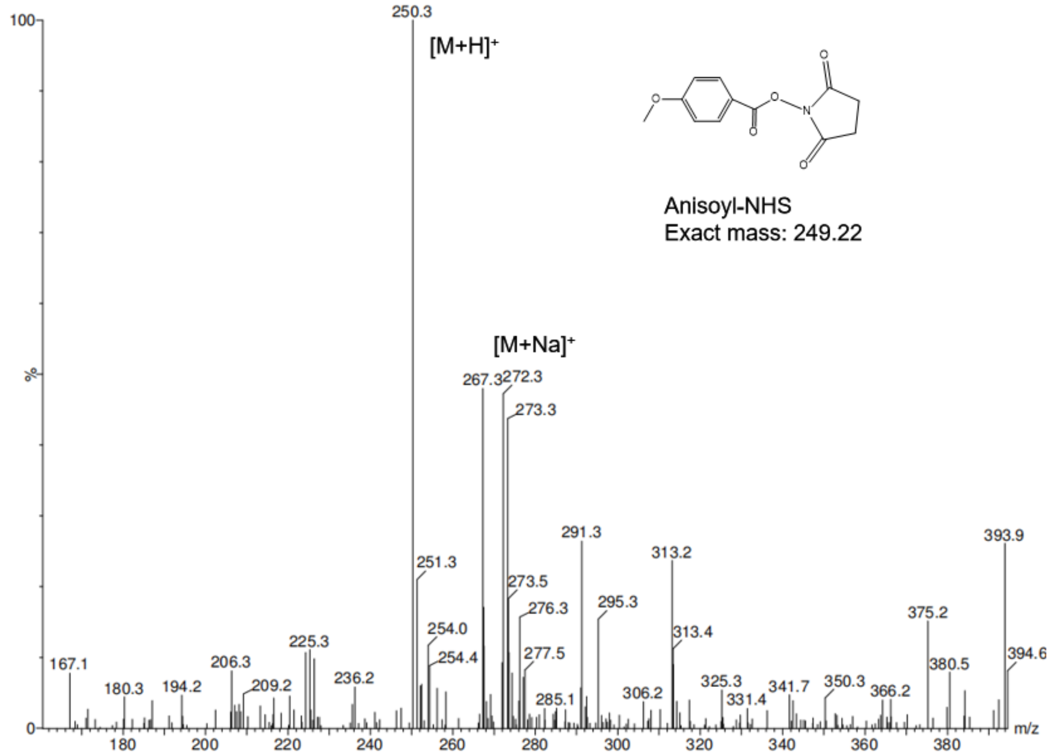

Figure S1. Mass spectrum of anisoyl-NHS.

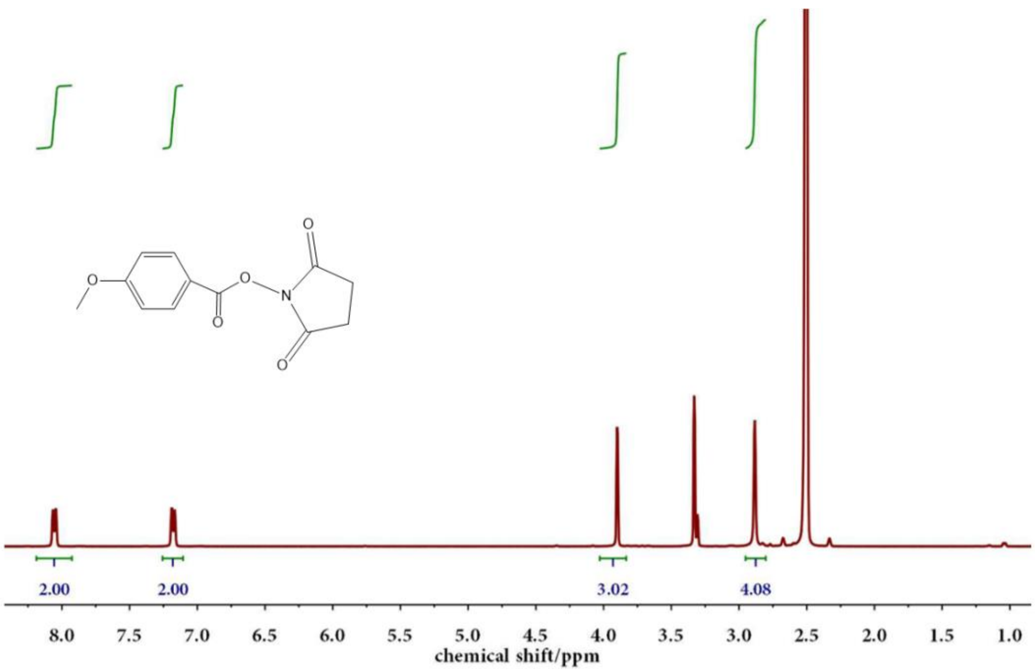

Figure S2. ^{1}H-NMR spectrum of anisoyl-NHS in DMSO-d_6.

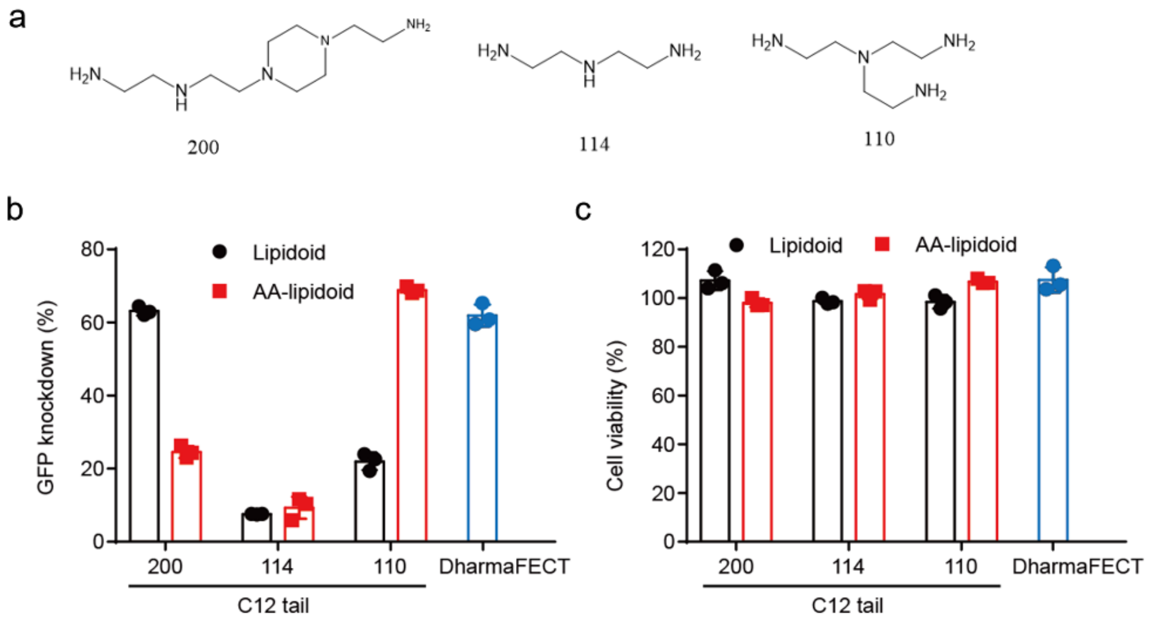

Figure S3. Pilot *in vitro* screening of three representative polyamine cores with C12 epoxide tail. (a) Piperazine derivative 200, linear amine 114 and branched amine 110. (b) GFP knockdown at 48 h post-treatment with 50 nM siGFP (n = 3/group). (c) Cell viability at 48 h post-treatment (n = 3/group). Data are presented as mean ± SD.

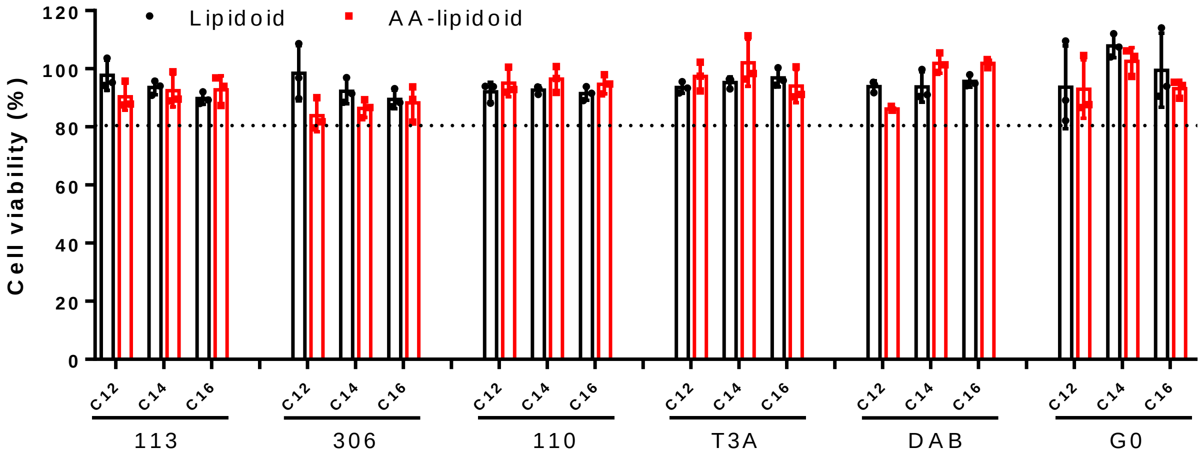

Figure S4. Cell viability at 48 h post-treatment (n = 3/group). Cell viabilities were above 80% for all LNPs. Data are presented as mean ± SD.

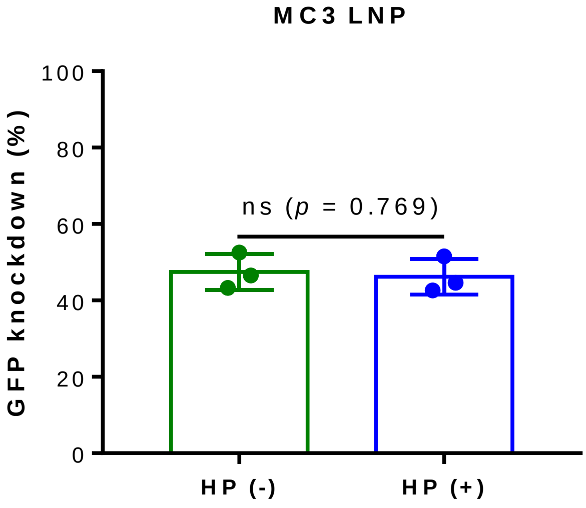

Figure S5. MC3 LNP-mediated GFP knockdown (n = 3/group). Activated 3T3-GFP fibroblasts were pre-treated with or without 30 μM haloperidol (HP) for 2 h before GFP siRNA-loaded MC3 LNP (50 nM) was used to treat cells for another 48 h. No obvious loss of silencing activity was observed after HP treatment. Data are presented as mean ± SD. ns, not significant. Data are analyzed by two-sided *t* test.

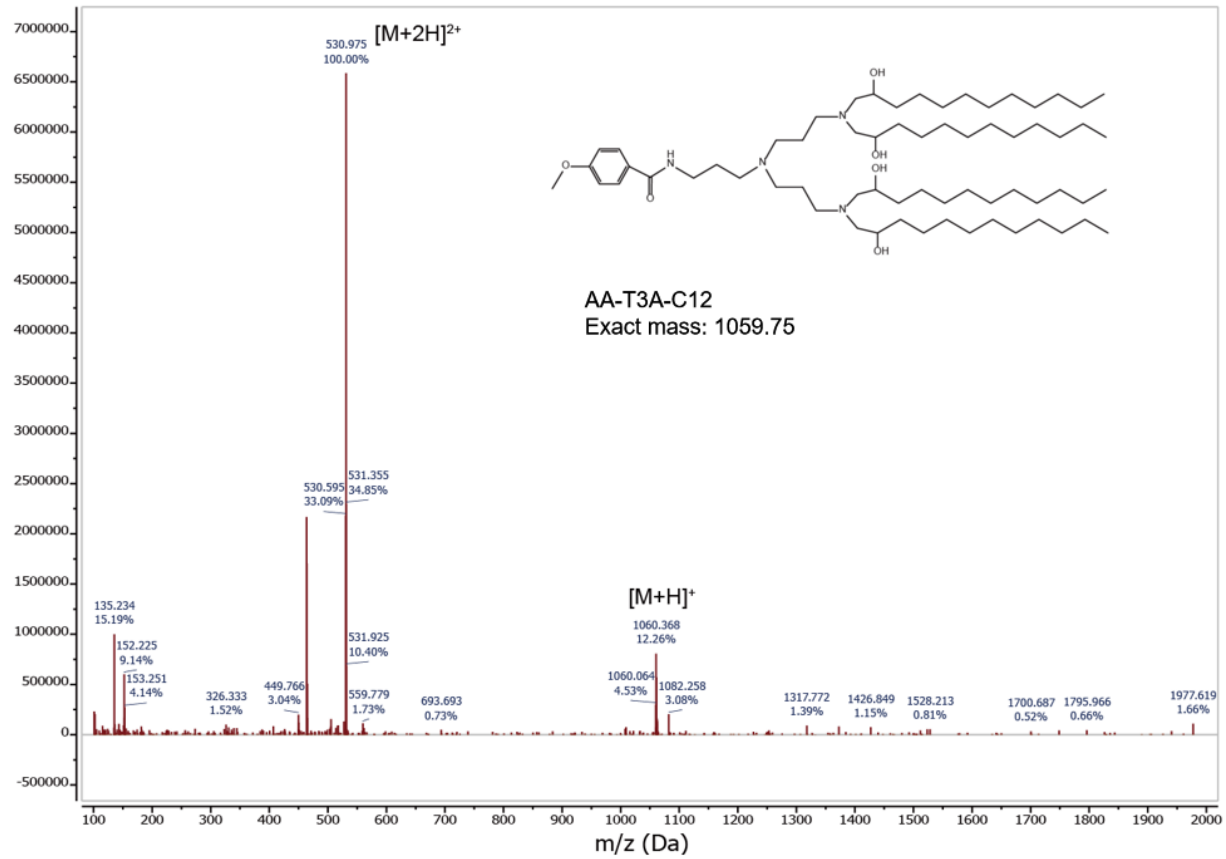

Figure S6. Mass spectrum of AA-T3A-C12.

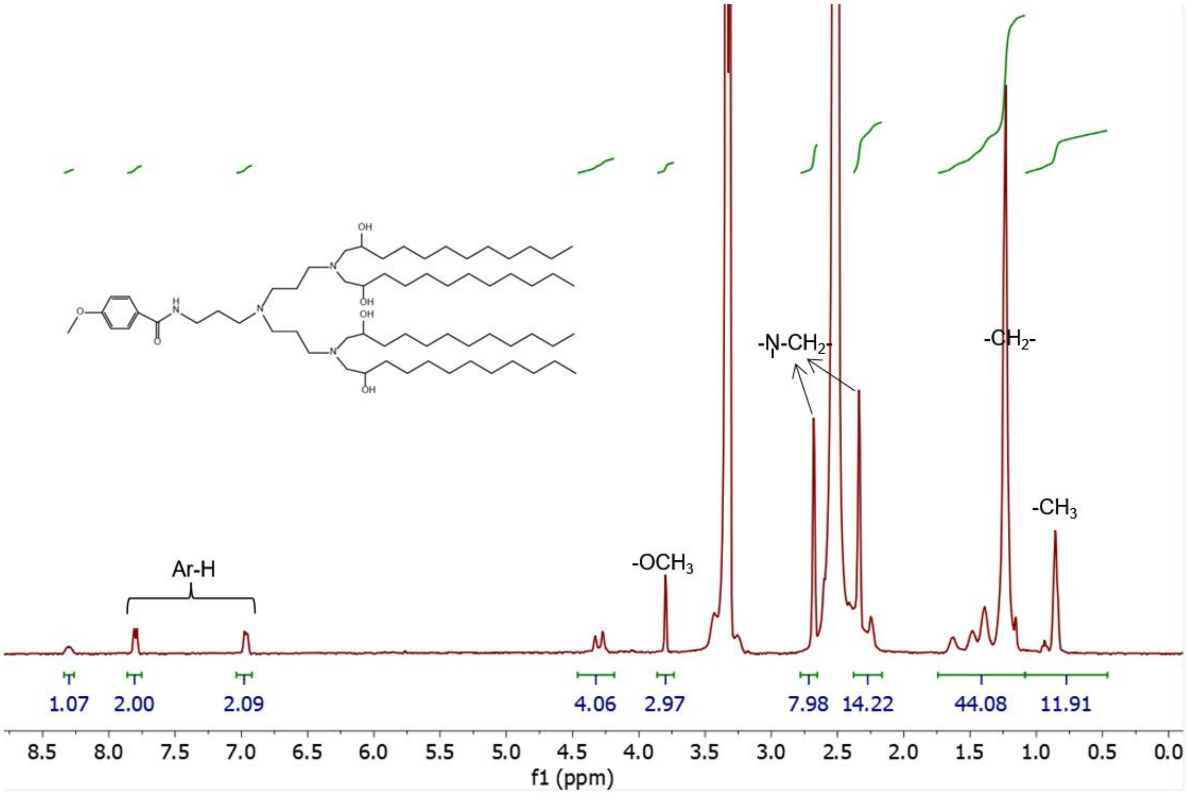

Figure S7. ^{1}H-NMR spectrum of AA-T3A-C12 in DMSO-d_6. The ^{1}H-NMR spectrum of AA-T3A-C12 showed characteristic peaks of anisamide, T3A core and epoxide tail.

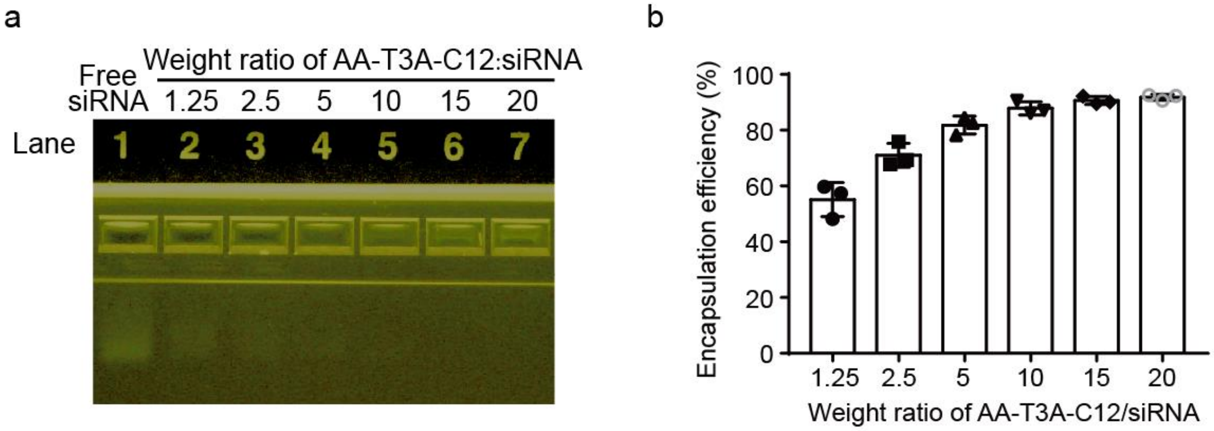

Figure S8. Optimization of the weight ratio of AA-T3A-C12 to siRNA. (a) Gel retardation assay. LNPs with different weight ratios of AA-T3A-C12:siRNA were analyzed by 1% agarose gel electrophoresis. 500 ng siRNA was loaded into each lane. Barely no free siRNA was observed when the weight ratio of AA-T3A-C12:siRNA was above 5:1. (b) RiboGreen RNA assay (n = 3/group). The siRNA encapsulation efficiency reached a plateau of ~90% when the weight ratio of AA-T3A-C12:siRNA was 10:1. Data are presented as mean ± SD.

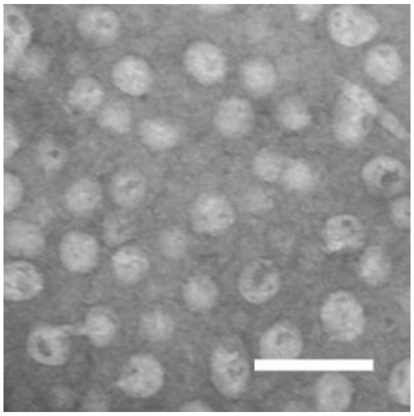

Figure S9. A representative TEM image of empty AA-T3A-C12 LNP from three independent experiments. Scale bar, 100 nm.

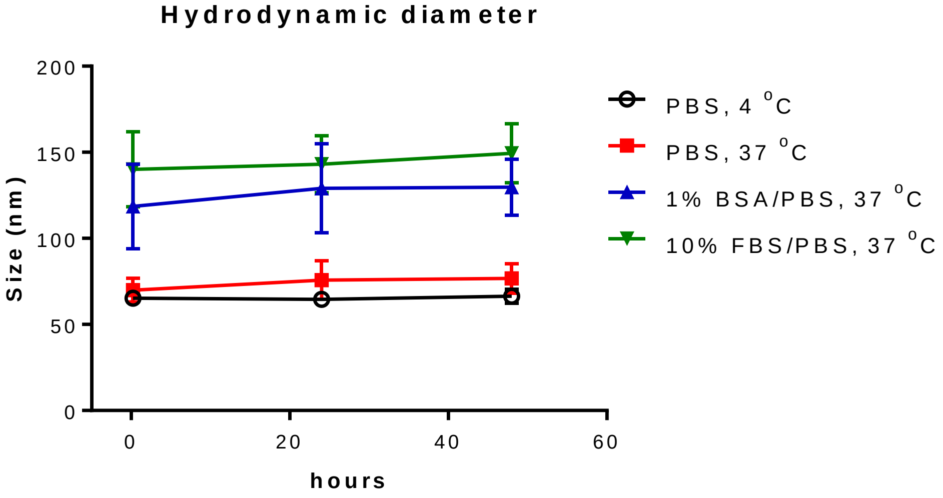

Figure S10. Hydrodynamic sizes of AA-T3A-C12/siRNA LNP (n = 3/group). 1% bovine serum albumin (BSA) and 10% FBS were prepared in 1×PBS and filtered through 0.22 μm filters to remove large protein aggregates. LNPs were then diluted in 1×PBS, 1% BSA/PBS or 10% FBS/PBS and incubated at the indicated temperature for 48 h. The average hydrodynamic size was determined by DLS. The absorption of protein on LNPs increased the size, but large LNP aggregates (> 200 nm) were not observed. Data are presented as mean ± SD.

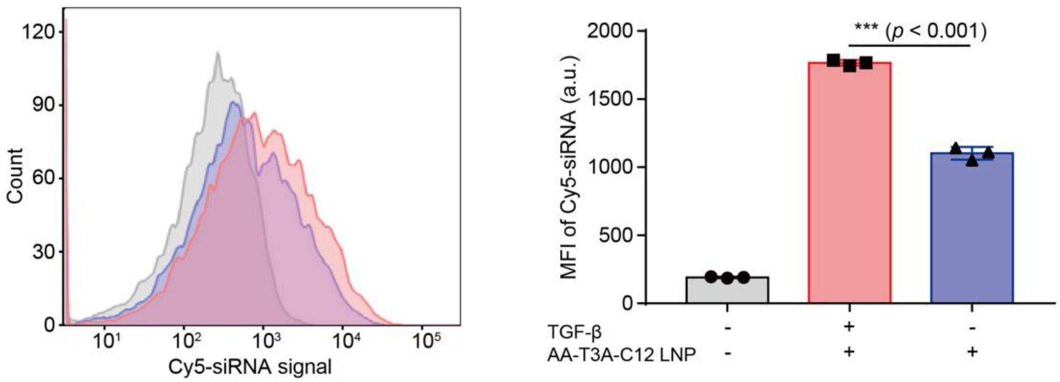

Figure S11. Flow cytometry analysis of cellular uptake of Cy5-siRNA-loaded AA-T3A-C12 LNP in 3T3 fibroblasts with or without TGF-β stimulation (representative dataset from n = 3/group). Data are presented as mean ± SD. ***p < 0.001. Data are analyzed by one-way ANOVA with Tukey's correction.

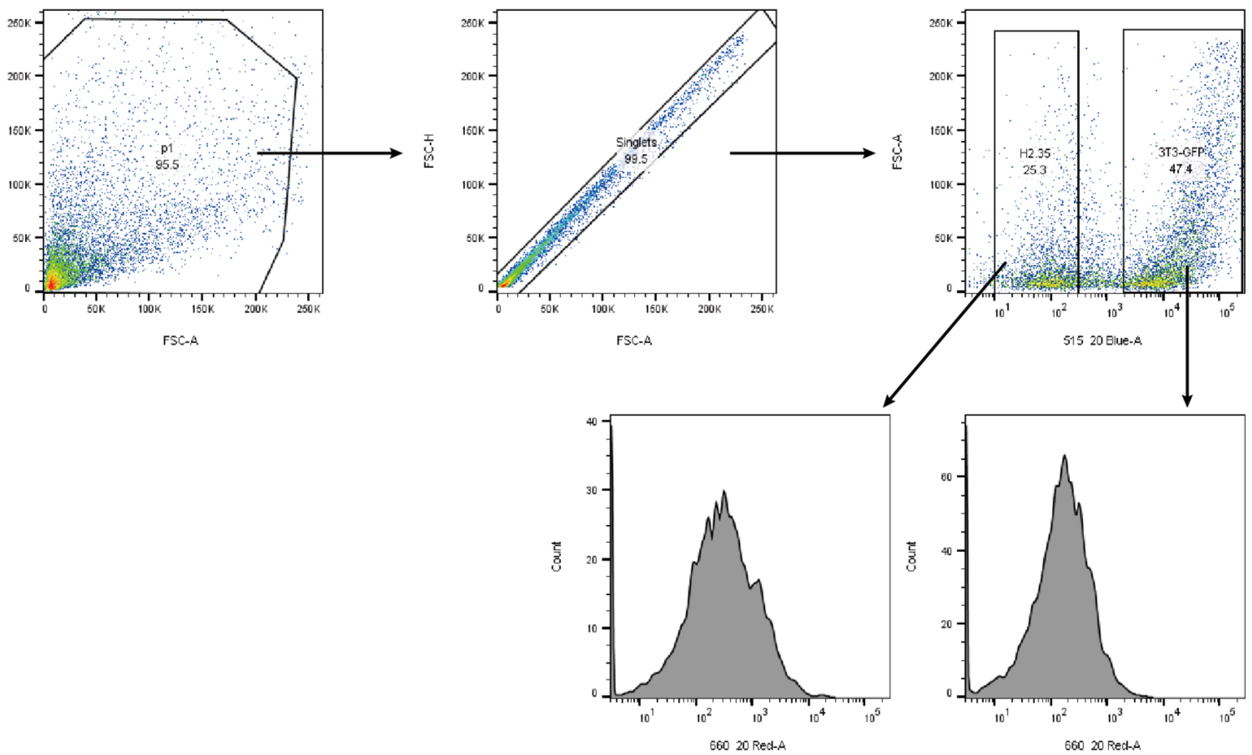

Figure S12. Flow cytometry gating strategy of Figure 3C. GFP-positive 3T3-GFP fibroblasts and GFP-negative H2.35 hepatocytes were gated to obtain Cy5-siRNA signal inside cells.

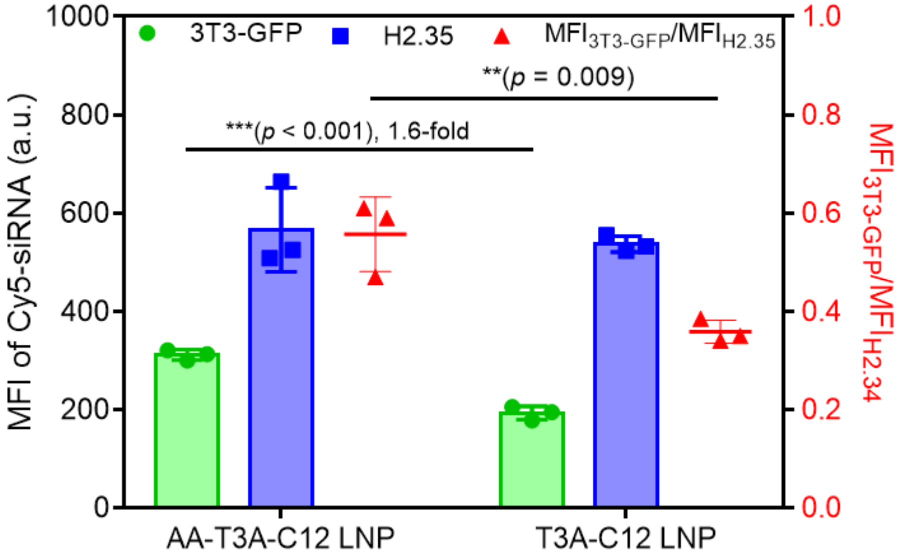

Figure S13. Flow cytometry analysis of competitive cellular uptake of Cy5-siRNA-loaded AA-T3A-C12 LNP or T3A-C12 LNP in a fibroblast/hepatocyte (3T3-GFP/H2.35) co-culture environment (representative dataset from n = 3/group). The mean fluorescence intensity ratio between fibroblast and hepatocyte ($MFI_{3T3\text{-}GFP}/MFI_{H2.35}$) was calculated to indicate the preferential uptake of LNPs by fibroblasts over hepatocytes. The $MFI_{3T3\text{-}GFP}/MFI_{H2.35}$ of AA-T3A-C12 LNP was 0.56, which was significantly higher than 0.36 of T3A-C12 LNP. Data are presented as mean ± SD. $^{**}p < 0.01$; $^{***}p < 0.001$. Data are analyzed by one-way ANOVA with Tukey's correction.

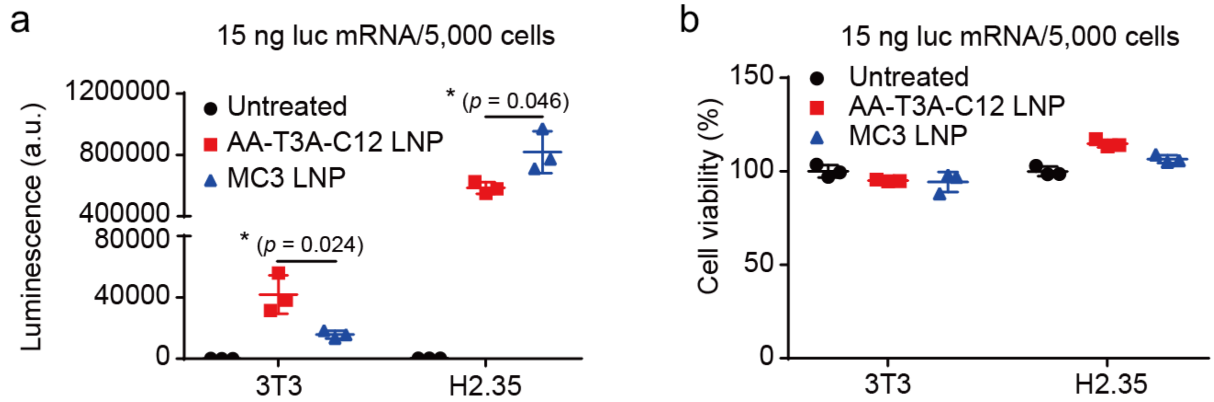

Figure S14. *In vitro* luciferase (luc) mRNA delivery by LNPs. (a) AA-T3A-C12 LNP- and MC3 LNP-mediated luc mRNA delivery in activated 3T3 fibroblasts and H2.35 hepatocytes (n = 3/group). 15 ng of luc mRNA was used to treat 5,000 cells per well for 24 h. (b) Cell viability at 24 h post-treatment (n = 3/group). Data are presented as mean ± SD. $*p < 0.05$. Data are analyzed by one-way ANOVA with Tukey's correction.

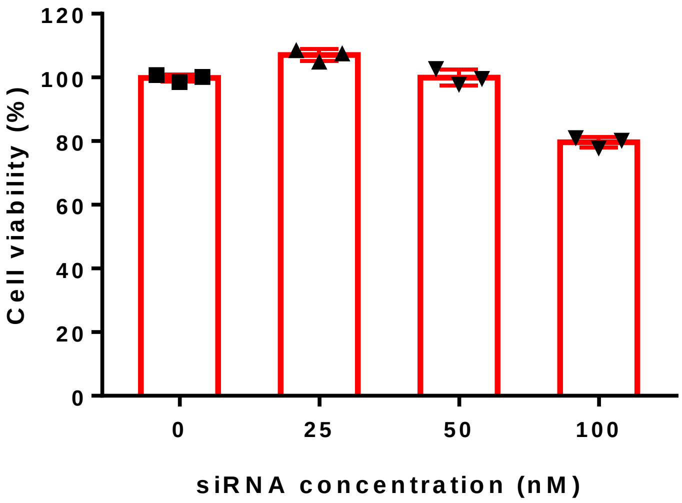

Figure S15. Cell viability after treatment with AA-T3A-C12/siGFP LNP for 48 h (n = 3/group). Cell viability was above or approximately 80% at the siRNA dose tested. Data are presented as mean ± SD.

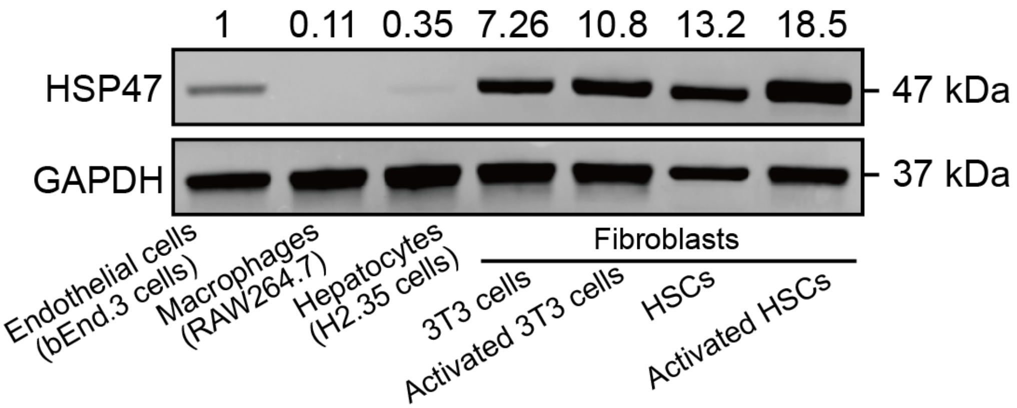

Figure S16. Western blot analysis of HSP47 expression in different cell types. Activated 3T3 cells and HSCs were obtained by stimulation with 10 ng/ml of TGF-β for 24 h. GAPDH was used as an internal control. HSP47 expression was normalized to endothelial cells. HSP47 expression was much higher in fibroblasts (primary HSCs and 3T3 cells) than other cell types. Moreover, activated fibroblasts further up-regulated HSP47 expression. A representative result is presented from three biologically independent experiments.

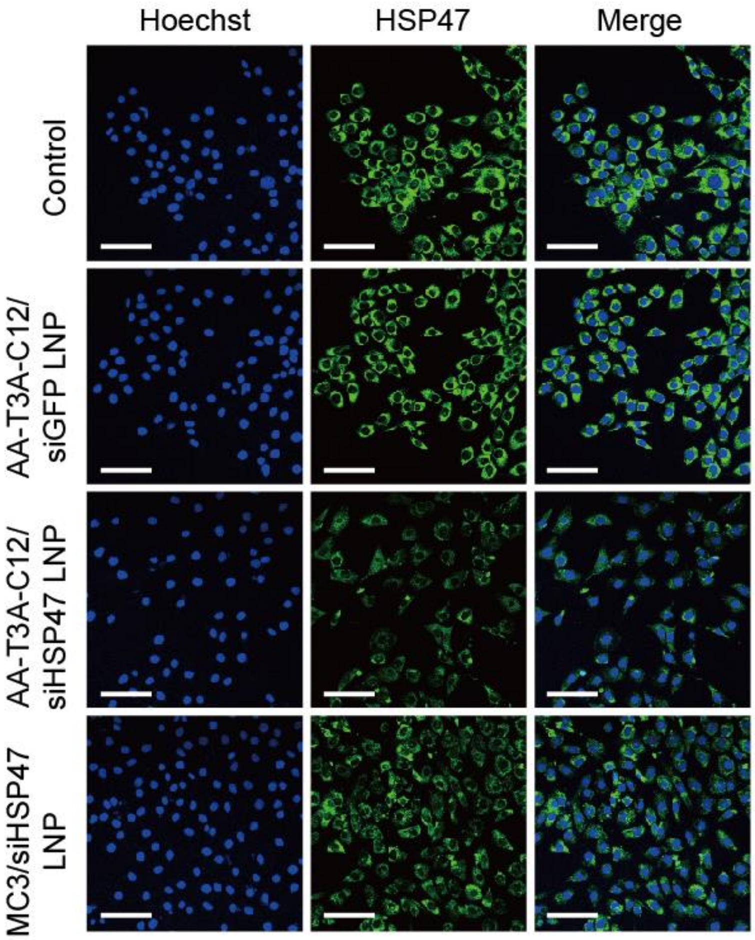

Figure S17. Immunofluorescence staining of HSP47 in LNP-treated activated 3T3 fibroblasts. A representative result is presented from three biologically independent experiments. Scale bar: 100 μm.

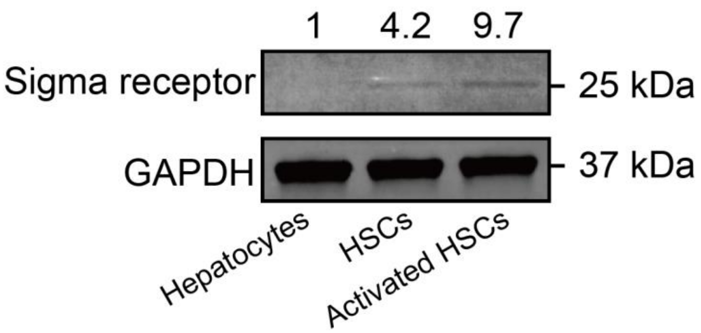

Figure S18. Western blot analysis of sigma receptor expression in immortalized primary hepatocytes H2.35, primary HSCs and activated primary HSCs. Activated HSCs were obtained by stimulation with 10 ng/ml of TGF-β for 24 h. GAPDH was used as an internal control. The expression of sigma receptor was normalized to hepatocytes. The expression of sigma receptor was much higher in HSCs than hepatocytes. Moreover, activated HSCs further up-regulated sigma receptors. A representative result is presented from three biologically independent experiments.

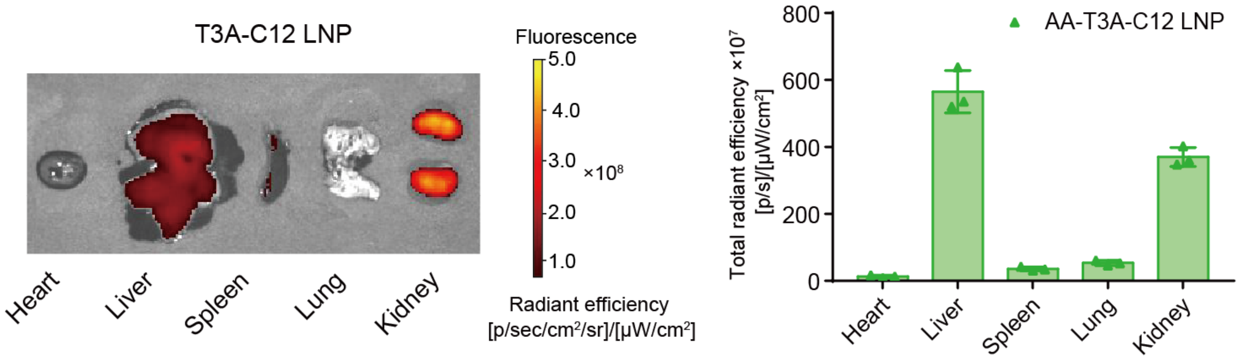

Figure S19. *Ex vivo* fluorescence imaging and signal quantification of major organs from T3A-C12 LNP/Cy5-siRNA treated mice (n = 3). Each mouse was i.v. injected with 5 µg of Cy5-siRNA. Data are presented as mean ± SD.

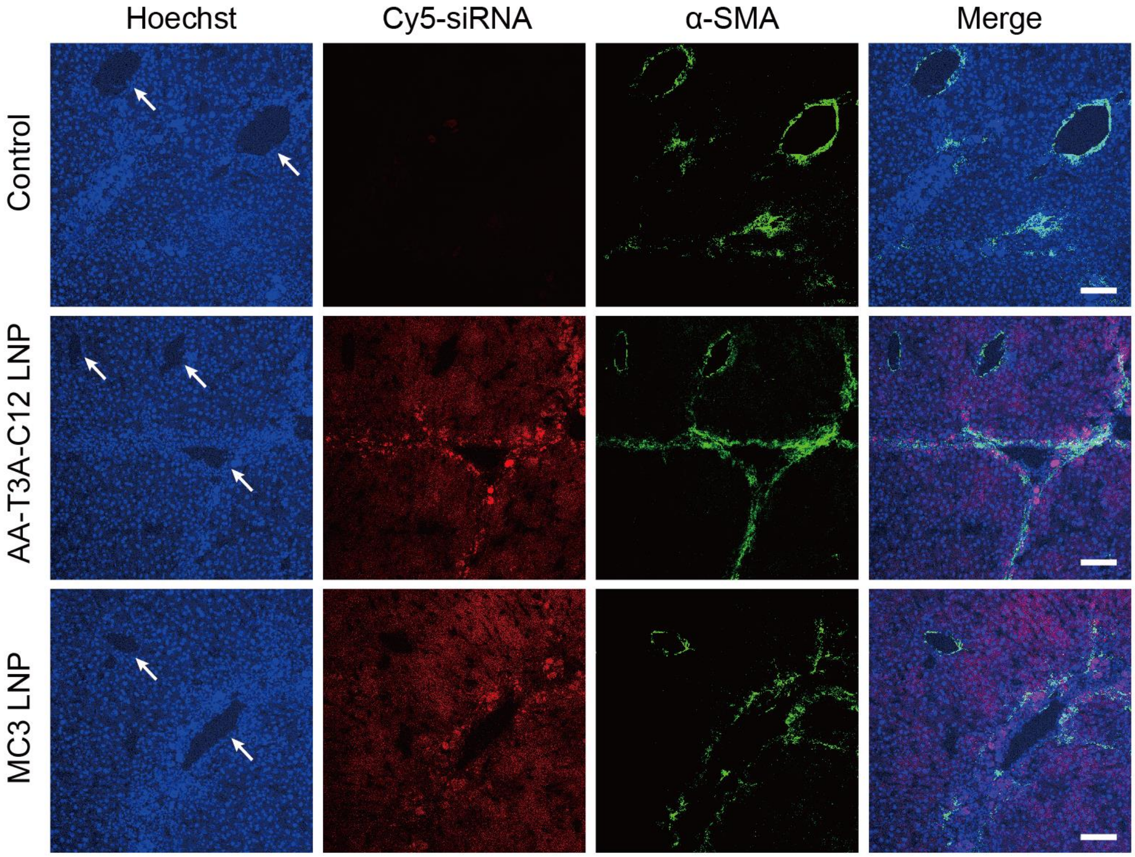

Figure S20. Representative confocal images of liver sections (representative dataset from n = 3/group). Fibrotic mice were i.v. injected with AA-T3A-C12 LNP/Cy5-siRNA or MC3 LNP/Cy5-siRNA at an siRNA dose of 5 μg/mouse. 1 h post-injection, mice were euthanized and livers were collected for immunofluorescence staining. Compared to MC3 LNP, more AA-T3A-C12 LNP co-localized with or were close to HSCs (α-SMA$^+$). Arrows indicate central veins of liver lobules. Scale bar: 100 μm.

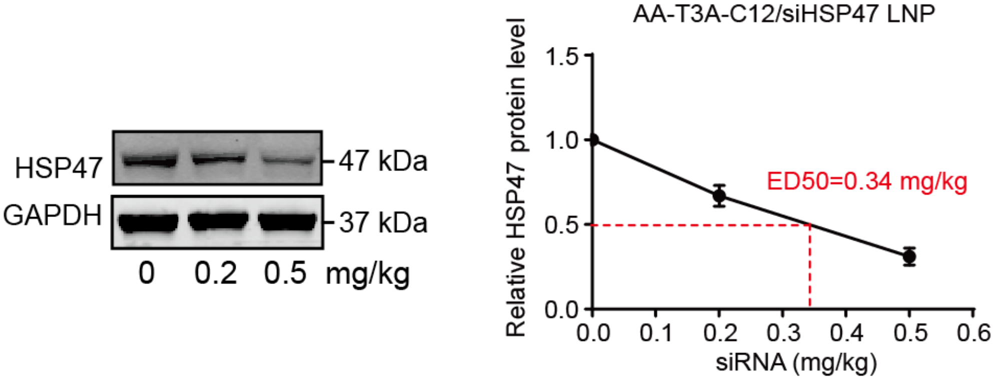

Figure S21. Western blot analysis of HSP47 expression in the liver (representative dataset from n = 3/group). Mice were i.p. injected with 20% CCl_4 (0.7 μl/g) in corn oil twice a week for 2 weeks and then i.v. injected with AA-T3A-C12/siHSP47 LNP at an siRNA dose of 5 μg/mouse (0.2 mg/kg) or 12.5 μg/mouse (0.5 mg/kg). Mice were euthanized 3 days after treatment and livers were harvested for western blot analysis. GAPDH was used as an internal control. Data are presented as mean ± SD.

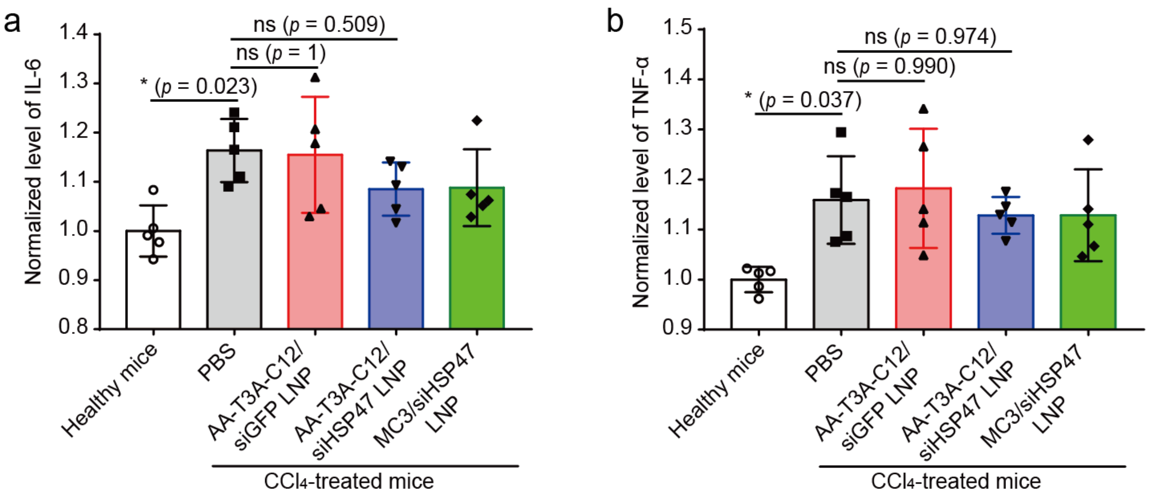

Figure S22. Normalized serum proinflammatory cytokine levels to healthy mice. (a) IL-6. (b) TNF-α. Data are presented as mean ± SD (n = 5/group). ns, not significant; *$p < 0.05$. Data are analyzed by one-way ANOVA with Tukey's correction.

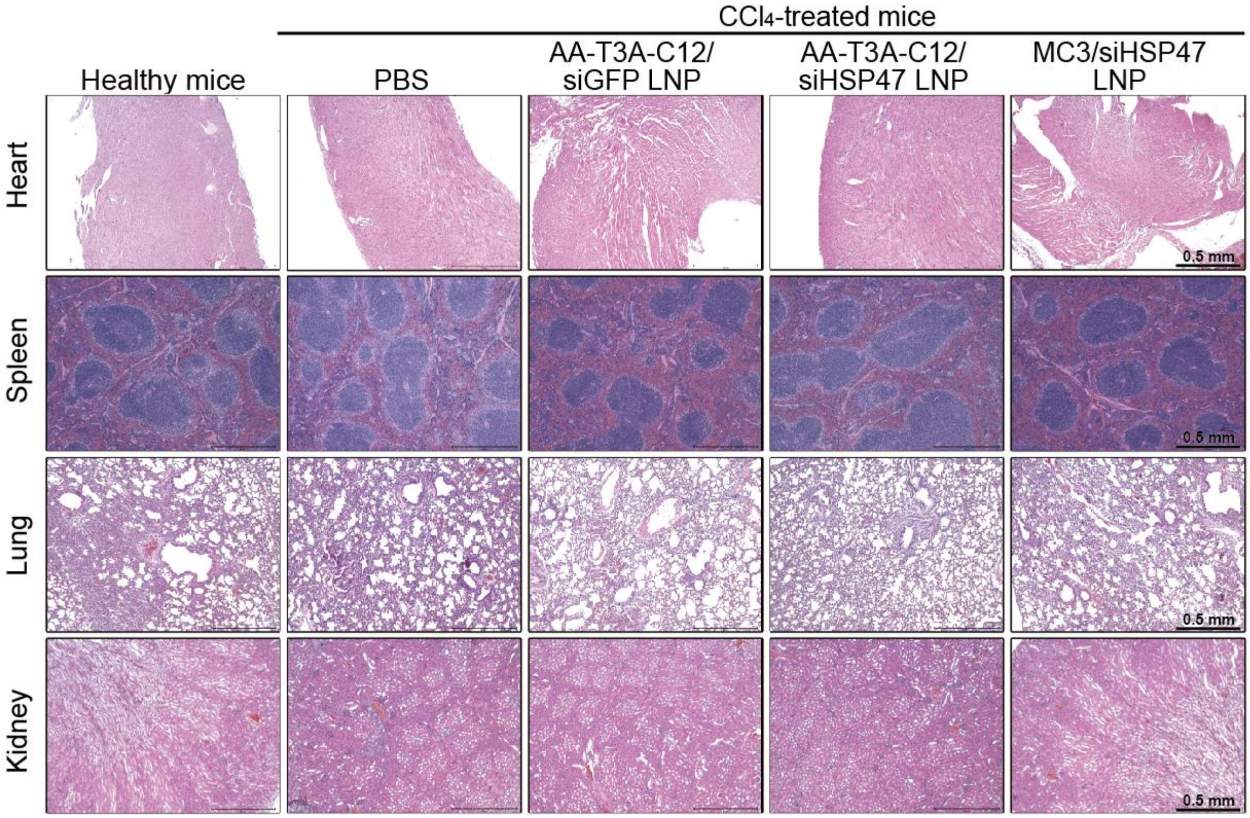

Figure S23. H&E staining of hearts, spleens, lungs and kidneys from each group (representative dataset from n = 5/group). No histological abnormality was observed for these organs.

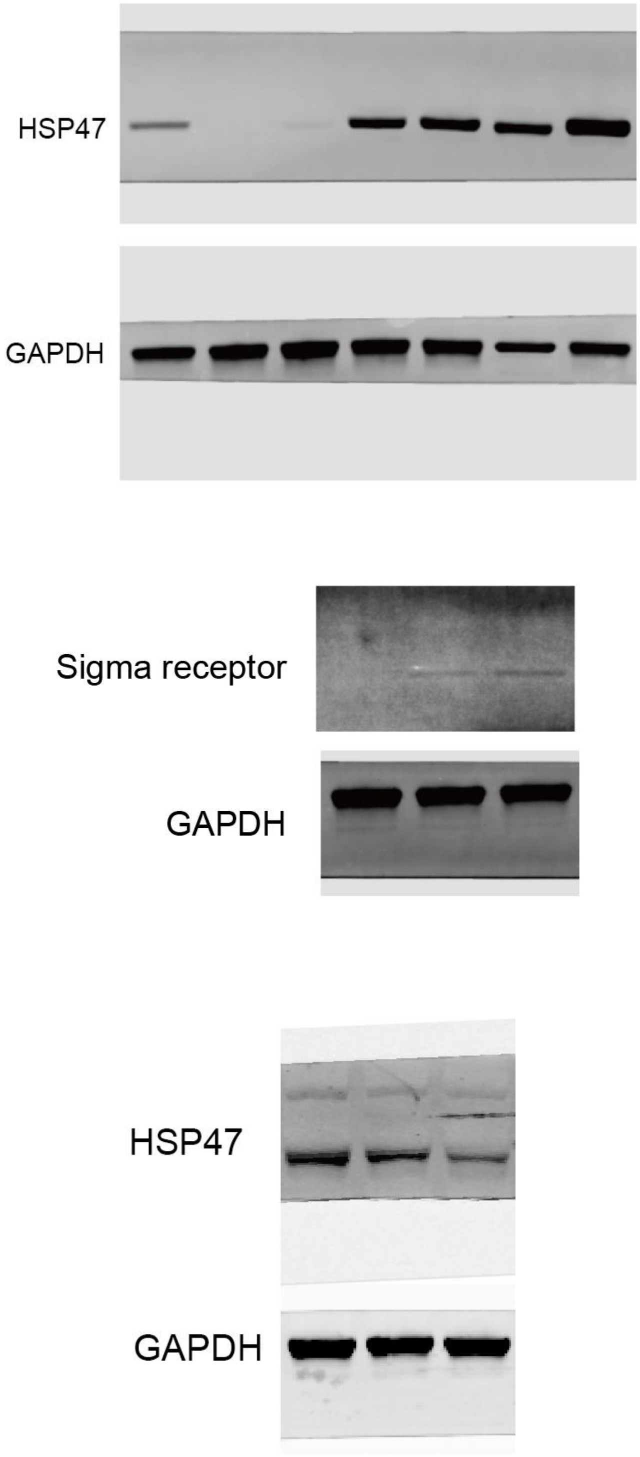

Figure S24. Uncropped blots of Fig. S16 (top), Fig. S18 (middle) and Fig. S21 (bottom).

Table S1. Characterization of LNPs.

**LNP**	**Diameter (nm)**	**PDI**	**Zeta potential (mV)**	**Encapsulation efficiency (%)**	**p*K*a**
Empty AA-T3A-C12 LNP	59.3 ± 2.5	0.021	-0.7 ± 0.9	n/a	n/a
AA-T3A-C12/siRNA LNP	65.6 ± 1.2	0.018	-1.1 ± 1.5	87.4 ± 3.8	5.72
T3A-C12/siRNA LNP	64.9 ± 1.8	0.034	-2.2 ± 1.8	33.6 ± 8.5	5.81
MC3/siRNA LNP	60.9 ± 3.4	0.090	-1.5 ± 2.2	90.1 ± 2.5	6.44[a]

[a]This data was obtained from the following publication: Jayaraman, M. *et al.* Maximizing the potency of siRNA lipid nanoparticles for hepatic gene silencing in vivo. *Angewandte Chemie* **51**, 8529-8533 (2012). n/a, not applicable.
