# Supplementary material for: Ligand-tethered lipid nanoparticles for targeted RNA delivery to treat liver fibrosis
Source: Nat Commun. 2023 Jan 17;14:75. doi: 10.1038/s41467-022-35637-z (PMC9845313; doi:10.1038/s41467-022-35637-z)
Supplement: Supplementary file 3 — Reporting Summary [file 41467_2022_35637_MOESM3_ESM.pdf]

nature portfolio

Corresponding author(s): Michael Mitchell

Last updated by author(s): Dec 8, 2022

# Reporting Summary

Nature Portfolio wishes to improve the reproducibility of the work that we publish. This form provides structure for consistency and transparency in reporting. For further information on Nature Portfolio policies, see our Editorial Policies and the Editorial Policy Checklist.

## Statistics

For all statistical analyses, confirm that the following items are present in the figure legend, table legend, main text, or Methods section.

| n/a | Confirmed | |
|---|---|---|
| ☐ | ☒ | The exact sample size (*n*) for each experimental group/condition, given as a discrete number and unit of measurement |
| ☐ | ☒ | A statement on whether measurements were taken from distinct samples or whether the same sample was measured repeatedly |
| ☐ | ☒ | The statistical test(s) used AND whether they are one- or two-sided<br>*Only common tests should be described solely by name; describe more complex techniques in the Methods section.* |
| ☒ | ☐ | A description of all covariates tested |
| ☐ | ☒ | A description of any assumptions or corrections, such as tests of normality and adjustment for multiple comparisons |
| ☐ | ☒ | A full description of the statistical parameters including central tendency (e.g. means) or other basic estimates (e.g. regression coefficient) AND variation (e.g. standard deviation) or associated estimates of uncertainty (e.g. confidence intervals) |
| ☐ | ☒ | For null hypothesis testing, the test statistic (e.g. *F*, *t*, *r*) with confidence intervals, effect sizes, degrees of freedom and *P* value noted<br>*Give P values as exact values whenever suitable.* |
| ☒ | ☐ | For Bayesian analysis, information on the choice of priors and Markov chain Monte Carlo settings |
| ☒ | ☐ | For hierarchical and complex designs, identification of the appropriate level for tests and full reporting of outcomes |
| ☒ | ☐ | Estimates of effect sizes (e.g. Cohen's *d*, Pearson's *r*), indicating how they were calculated |

*Our web collection on statistics for biologists contains articles on many of the points above.*

## Software and code

Policy information about availability of computer code

| | |
|---|---|
| Data collection | Zeiss LSM 710, PerkinElmer IVIS Spectrum, BD LSR II Flow Cytometer |
| Data analysis | Statistical analysis was performed on Graphpad Prism 7.0; Flow cytometry data were analyzed on FlowJo software package (Flowjo V10); Western blots were analyzed by ImageJ software; 1H NMR spectrum was analyzed by MestReNova x64; Confocol images were analyzed by Zen 2011 software. |

For manuscripts utilizing custom algorithms or software that are central to the research but not yet described in published literature, software must be made available to editors and reviewers. We strongly encourage code deposition in a community repository (e.g. GitHub). See the Nature Portfolio guidelines for submitting code & software for further information.

## Data

Policy information about availability of data

All manuscripts must include a data availability statement. This statement should provide the following information, where applicable:

- Accession codes, unique identifiers, or web links for publicly available datasets
- A description of any restrictions on data availability
- For clinical datasets or third party data, please ensure that the statement adheres to our policy

All data are available in the main text or the supplementary materials. Source data are provided with this paper.

## Human research participants

Policy information about studies involving human research participants and Sex and Gender in Research.

| | |
|---|---|
| Reporting on sex and gender | No human participants were involved in this study. |
| Population characteristics | No human participants were involved in this study. |
| Recruitment | No human participants were involved in this study. |
| Ethics oversight | No human participants were involved in this study. |

Note that full information on the approval of the study protocol must also be provided in the manuscript.

# Field-specific reporting

Please select the one below that is the best fit for your research. If you are not sure, read the appropriate sections before making your selection.

☒ Life sciences ☐ Behavioural & social sciences ☐ Ecological, evolutionary & environmental sciences

For a reference copy of the document with all sections, see nature.com/documents/nr-reporting-summary-flat.pdf

# Life sciences study design

All studies must disclose on these points even when the disclosure is negative.

| | |
|---|---|
| Sample size | No statistical methods were used to determine the sample size in the study. The sample sizes are clearly described in each figure legend. The sample size were determined by allowable error size, accuracy, resources, and need for statistical analysis (generally n>=3 throughout all the studies). |
| Data exclusions | No animals and/or data were excluded. |
| Replication | All experiments were repeated for at least three times and experimental findings were reproducible. |
| Randomization | For in vivo study, animal groups were randomized by body weight. For other experiments, all samples were randomly allocated into experimental groups, as there was no covariate in the study design. |
| Blinding | All the investigators were blinded to group allocation during data collection and analysis. |

# Reporting for specific materials, systems and methods

We require information from authors about some types of materials, experimental systems and methods used in many studies. Here, indicate whether each material, system or method listed is relevant to your study. If you are not sure if a list item applies to your research, read the appropriate section before selecting a response.

## Materials & experimental systems

| n/a | Involved in the study | |
|---|---|---|
| ☐ | ☒ | Antibodies |
| ☐ | ☒ | Eukaryotic cell lines |
| ☒ | ☐ | Palaeontology and archaeology |
| ☐ | ☒ | Animals and other organisms |
| ☒ | ☐ | Clinical data |
| ☒ | ☐ | Dual use research of concern |

## Methods

| n/a | Involved in the study | |
|---|---|---|
| ☒ | ☐ | ChIP-seq |
| ☐ | ☒ | Flow cytometry |
| ☒ | ☐ | MRI-based neuroimaging |

## Antibodies

| | |
|---|---|
| Antibodies used | IRDye 800CW donkey anti-mouse IgG secondary antibody (925-32212, LiCor), HSP47 antibody (NBP1-97491, Novus Biologicals), Alexa Fluor® 488 conjugated goat anti-mouse IgG (H+L), F(ab')2 fragment antibody (#4408, CST), Sigma Receptor antibody (#sc-137075, Santa Cruz), FITC-conjugated α-smooth muscle actin antibody (#F3777, Sigma) |
| Validation | All antibodies were verified by the supplier and each lot has been quality tested. All the antibodies used are from commercial sources |

| | |
|---|---|
| Validation | and have been validated by the vendors. Validation statements (species, application et al.) and data are available on the manufacturer's website. |

## Eukaryotic cell lines

Policy information about cell lines and Sex and Gender in Research

| | |
|---|---|
| Cell line source(s) | The murine NIH 3T3 fibroblast cell line was kindly provided by Prof. Andrew Tsourkas (University of Pennsylvania), who obtained it from ATCC (cat. CRL-1658). The NIH 3T3-GFP cell line was purchased from Cell Biolabs (cat. AKR-214). The murine H2.35 hepatocyte cell line was kindly provided by Prof. James M. Wilson (University of Pennsylvania), who obtained it from ATCC (cat. CRL-1995). The primary murine HSCs were obtained from Sciencell (cat. M5300-57). The murine bEnd.3 endothelial cell line (cat. CRL-2299) and the murine RAW264.7 macrophage cell line (cat. TIB-71) were obtained from ATCC. |
| Authentication | A short tandem repeat DNA profiling method was used to authenticate the cell lines and the results were compared with reference database. |
| Mycoplasma contamination | All cell lines were tested for mycoplasma contamination. No mycoplasma contamination was found. |
| Commonly misidentified lines (See ICLAC register) | No commonly misidentified cell lines were used. |

## Animals and other research organisms

Policy information about studies involving animals; ARRIVE guidelines recommended for reporting animal research, and Sex and Gender in Research

| | |
|---|---|
| Laboratory animals | Balb/c male mice (6-8 weeks age, 22-25 g body weight) were purchased from Jackson Laboratory and housed in a specific-pathogen-free animal facility at ambient temperature (22 ± 2 °C), air humidity 40%–70% and 12-h dark/12-h light cycle. |
| Wild animals | No wild animal was used in this study. |
| Reporting on sex | Only male mice were used in this study, since female hormones can affect the fibrosis progression resulting in variations. |
| Field-collected samples | The study did not involve samples collected from field. |
| Ethics oversight | All animal experiment protocols were reviewed and approved by the institutional animal care and use committee of the University of Pennsylvania. |

Note that full information on the approval of the study protocol must also be provided in the manuscript.

## Flow Cytometry

### Plots

Confirm that:

☒ The axis labels state the marker and fluorochrome used (e.g. CD4-FITC).

☒ The axis scales are clearly visible. Include numbers along axes only for bottom left plot of group (a 'group' is an analysis of identical markers).

☒ All plots are contour plots with outliers or pseudocolor plots.

☒ A numerical value for number of cells or percentage (with statistics) is provided.

### Methodology

| | |
|---|---|
| Sample preparation | Cells were trypsinized. Single-cell suspensions were obtained and and then analyzed by flow cytometry for GFP or Cy5 signal. |
| Instrument | BD LSR II |
| Software | FlowJo software package (Flowjo V10) |
| Cell population abundance | The absolute cells >= 10000 were analyzed for fluorescent intensity in the defined gate. |
| Gating strategy | In general, cells were first gated on FSC/SSC. Singlet cells were gated using FSC-H and FSC-A. Dead cells were then excluded and further surface and intracellular antigen gating was performed on the live cell population (See supplementary infomation). |

☒ Tick this box to confirm that a figure exemplifying the gating strategy is provided in the Supplementary Information.
